# Exploring the Differences in Molecular Mechanisms and Key Biomarkers Between Membranous Nephropathy and Lupus Nephritis Using Integrated Bioinformatics Analysis

**DOI:** 10.3389/fgene.2021.770902

**Published:** 2022-01-03

**Authors:** Zhaocheng Dong, Haoran Dai, Wenbin Liu, Hanxue Jiang, Zhendong Feng, Fei Liu, Qihan Zhao, Hongliang Rui, Wei Jing Liu, Baoli Liu

**Affiliations:** ^1^ Beijing Hospital of Traditional Chinese Medicine, Capital Medical University, Beijing, China; ^2^ Renal Research Institution of Beijing University of Chinese Medicine, and Key Laboratory of Chinese Internal Medicine of Ministry of Education and Beijing, Dongzhimen Hospital, Beijing University of Chinese Medicine, Beijing, China; ^3^ Shunyi Branch, Beijing Traditional Chinese Medicine Hospital, Beijing, China; ^4^ Beijing University of Chinese Medicine, Beijing, China; ^5^ Beijing Chinese Medicine Hospital Pinggu Hospital, Beijing, China; ^6^ Capital Medical University, Beijing, China

**Keywords:** membranous nephropathy, lupus nephritis, integrated bioinformatics analysis, biomarker, differential gene analysis

## Abstract

**Background:** Both membranous nephropathy (MN) and lupus nephritis (LN) are autoimmune kidney disease. In recent years, with the deepening of research, some similarities have been found in the pathogenesis of these two diseases. However, the mechanism of their interrelationship is not clear. The purpose of this study was to investigate the differences in molecular mechanisms and key biomarkers between MN and LN.

**Method:** The expression profiles of GSE99325, GSE99339, GSE104948 and GSE104954 were downloaded from GEO database, and the differentially expressed genes (DEGs) of MN and LN samples were obtained. We used Gene ontology (GO) and Kyoto Encyclopedia of Genes and Genomes (KEGG) for enrichment analysis of DEGs. A protein-protein interaction (PPI) network of DEGs was constructed using Metascape. We filtered DEGs with NetworkAnalyst. Finally, we used receiver operating characteristic (ROC) analysis to identify the most significant DEGs for MN and LN.

**Result:** Compared with LN in the glomerulus, 14 DEGs were up-regulated and 77 DEGs were down-regulated in MN. Compared with LN in renal tubules, 21 DEGs were down-regulated, but no up-regulated genes were found in MN. According to the result of GO and KEGG enrichment, PPI network and Networkanalyst, we screened out six genes (IFI6, MX1, XAF1, HERC6, IFI44L, IFI44). Interestingly, among PLA2R, THSD7A and NELL1, which are the target antigens of podocyte in MN, the expression level of NELL1 in MN glomerulus is significantly higher than that of LN, while there is no significant difference in the expression level of PLA2R and THSD7A.

**Conclusion:** Our study provides new insights into the pathogenesis of MN and LN by analyzing the differences in gene expression levels between MN and LN kidney samples, and is expected to be used to prepare an animal model of MN that is more similar to human.

## Introduction

MN is one of the pathological types of primary glomerular diseases (Couser, 2017). It is characterized by diffuse glomerular lesions, thickening of capillary walls, and deposition of autoantibodies *in situ* immune complexes directly with glomerular antigens under the epithelium. Nephrotic syndrome occurs in approximately 80% of patients with membranous nephropathy. In symptoms, MN patients often do not present gross hematuria. Some patients can achieve spontaneous remission (Stangou et al., 2019). With the increase of its incidence, more and more discussions on its pathogenesis have been made. Among them, the alveolar extracellular trap formation hypothesis proposed by Liu et al. is the most interesting. It is believed that the incidence of MN is related to air pollution (Liu et al., 2019). The most common pathogenic antigen of this disease, the M-type phospholipase A2 receptor (PLA2R), also happens to be expressed on alveolar macrophages and neutrophils (Silliman et al., 2002; Granata et al., 2005). Therefore, due to the stimulation of polluted air, neutrophils and macrophages in alveoli form extracellular traps, enabling PLA2R to be recognized as its own antigen. However, these extracellular traps, which do not contain cytoskeletal proteins, are made of nucleic acid material (Goggs et al., 2020). The main body of these nucleic acids is DNA. If extracellular traps are recognized by antigen-presenting cells, they should not only deliver PLA2R, but also Myeloperoxidase (MPO), histones, and even DNA, which are components of extracellular traps. In systemic lupus erythematosus (SLE), autoantibodies directly target nuclear antigens, including DNA (dsDNA and ssDNA), histones and ribonucleoproteins (Thong and Olsen, 2017).

SLE is a multisystem disease characterized by systemic symptoms, musculoskeletal and visceral inflammation (Bakshi et al., 2018). Most cases of the disease have a slow onset. The etiology of SLE is unknown. A large number of studies have shown that the incidence of SLE is related to genetic factors, endocrine disorders, infection, immune abnormalities and environmental factors (Leffers et al., 2019; Catalina et al., 2020). In terms of environmental factors, smoking history is a risk factor for SLE. Smokers are more likely to develop SLE than non-smokers (Montes et al., 2016). Similarly, air pollution also contributes to the onset of SLE (Jung et al., 2019; Cakmak et al., 2021). In terms of immunity, SLE has a variety of immune abnormalities, such as the production of autoantibodies, immune complexes, autoreactive or inflammatory T cells and inflammatory cytokines. These abnormalities activate and amplify the inflammatory response, causing organ damage, leading to the onset and exacerbation of the disease (Aringer et al., 2021; Hansen et al., 2021). LN is the most common organ complication in SLE. Its clinical manifestations are hematuria, proteinuria, nephrotic syndrome, acute or chronic renal failure, etc.

Therefore, the pathogenesis of extracellular trap formation in alveolar should not only induce MN, but also may induce LN (Frangou et al., 2019). Moreover, more and more peer researchers have recognized this pathogenesis of MN. In addition, there are many differences between MN and LN. Although both are autoimmune diseases, renal pathology of LN shows glomerular intrinsic cell proliferation and circulating inflammatory cell infiltration, while MN rarely shows these pathological changes (Cuitino et al., 2019; Motavalli et al., 2019; Sung and Fu, 2020). The pathogenic antibody of MN is IgG, and IgG4 is the main type (Liu et al., 2020). IgA, IgM and IgG were found in LN kidney (Song et al., 2020). Although complement is involved in the pathogenesis of both MN and LN, the activation of MN complement is only thought to depend on the alternative and lectin pathways (van de Logt et al., 2019; Zhang et al., 2019; Haddad et al., 2021). Therefore, in order to discover the differences between the molecular mechanisms of these two diseases, as well as the key biomarkers that can predict and identify them, we explored these issues using comprehensive bioinformatics analysis.

## Materials and Methods

### Inclusion and Exclusion Criteria and Data Collection

To investigate the differences in molecular mechanisms and key biomarkers between MN and LN, we included the series of patients’ glomerulus or renal tubules. These gene expression profiles must have both MN and LN. These gene expression profiles must be based on human samples. Because of differences in gene expression between glomerular and tubular samples, we excluded series that tested whole kidney samples. We excluded the gene expression profiles that studied non-coding RNA. We excluded samples or series with progressive CKD. To sum up, we searched the GEO (Gene Expression Omnibus) database (https://www.ncbi.nlm.nih.gov/geo/) using the following keywords: “membranous nephropathy” AND “lupus” AND “*Homo sapiens*” AND “Expression profiling by array.” Then, the search results are filtered according to the criteria.

### Identification of DEGs

After a systematic review, four gene expression profiles (GSE99325, GSE99339, GSE104948 and GSE104954) were collected for analysis. The gene expression profiles of glomerular samples were GSE99339 and GSE104948, and those of renal tubule samples were GSE99325 and GSE104954. Then, the R package limma was used for differential expression gene (DEG) analysis. For GSE99325, GSE104948 and GSE104954, we perceived *p* < 0.05 and a |log(FC, fold change)| >1 to be statistically significant for the DEGs, and logFC ≥1 and logFC ≤ -1 were used to indicate upregulated and downregulated DEGs, respectively. For the maximum value in GSE99339 data is less than 1 and the minimum value is greater than -1, we perceived *p* < 0.05 and a |log(FC, fold change)| >0.2 to be statistically significant for the DEGs, and logFC ≥0.2 and logFC ≤ -0.2 were used to indicate upregulated and downregulated DEGs, respectively. In order to identify signifcant DEGs, the Venn online tool (http://bioinformatics.psb.ugent.be/webtools/Venn/) was used to draw a Venn map, and overlapping DEGs were retained for further analysis (Zhao et al., 2021). Volcano maps were drawn using the volcano plotting tool (http://soft.sangerbox.com/).

### Functional Enrichment Analysis and PPI Network Construction

To functionally annotate DEGs identifed by the aforementioned comparison groups, annotation and visualization of GO terms was used by WebGestalt (WEB-based Gene SeT AnaLysis Toolkit) (http://webgestalt.org/) and metascape (http://metascape.org/gp/index.html#/main/step1). The DEGs were then still introduced into the WebGestal for KEGG pathway analysis. We also constructed the protein-protein interaction (PPI) network of DEGs with metascape. After that, molecular complex detection (MCODE) algorithm was applied to identify the tightly connected neighborhood of the protein. Each MCODE network is assigned a unique color. GO enrichment analysis is performed on each MCODE network to give meaning to network components. And the hub genes were screened.

### Data Integration and DEGs Screening

We used the “Multiple Gene Expression Table” in Networkanalyst (https://www.networkanalyst.ca/) to integrate the data of GSE99339 and GSE104948 into the glomerular gene expression profile. The data of GSE99325 and GSE104954 were integrated into the gene expression profile of renal tubules, and the study batch effect was adjusted. After adjusting the batch effect, the gene expression data of the combined glomerular and renal tubule samples were obtained, and principal component analysis (PCA) plot and Density plot were plotted respectively (Zhou G et al., 2019). From the glomerular and renal tubule samples, we screened out significantly different genes from the common DEGs in the two groups of data, based on | Combined Tstat | > 200. At the same time, we used the above data sets to draw heat maps (http://soft.sangerbox.com/). Through the above analysis, we selected the most representative several DEGs, and used the Omicshare (https://www.omicshare.com/tools/) to draw violin plots, to visually display the difference in DEGs expression between MN and LN.

### Receiver Operator Characteristic Analysis

Based on the DEGs obtained from the above analysis, we performed multivariate modeling with SPSS 20.0 in combination with selected genes to identify key biomarkers with high sensitivity and specificity for the identification of MN and LN. The ROC curve was plotted and the area under curve (AUC) was calculated to evaluate the performance of each key biomarker. AUC>0.95 or <0.05 indicates that the model has a good fitting effect.

## Result

### Identifcation and Analysis of DEGs

**FIGURE 1:**
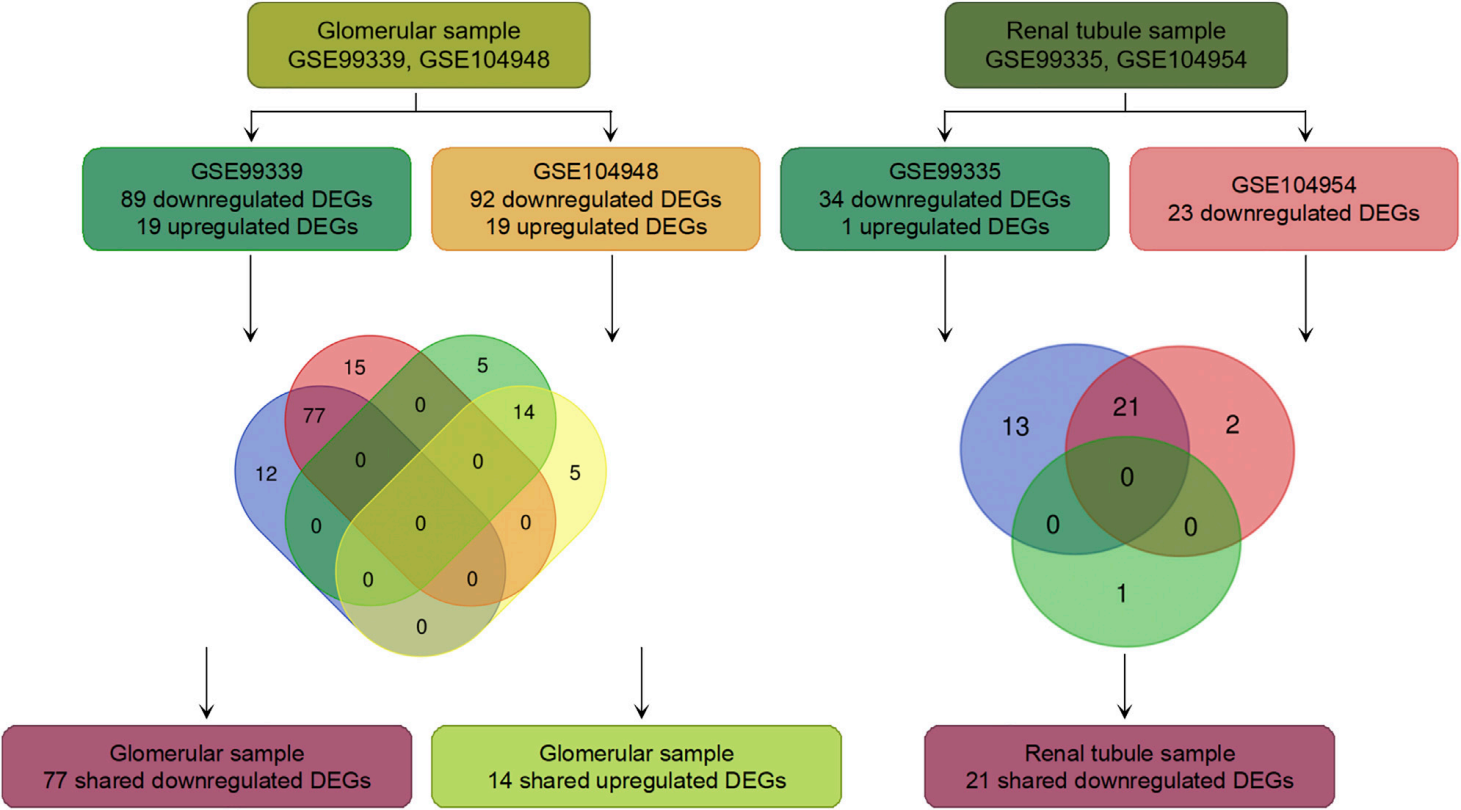
Roadmap of the approach and summarized findings.

**FIGURE 2:**
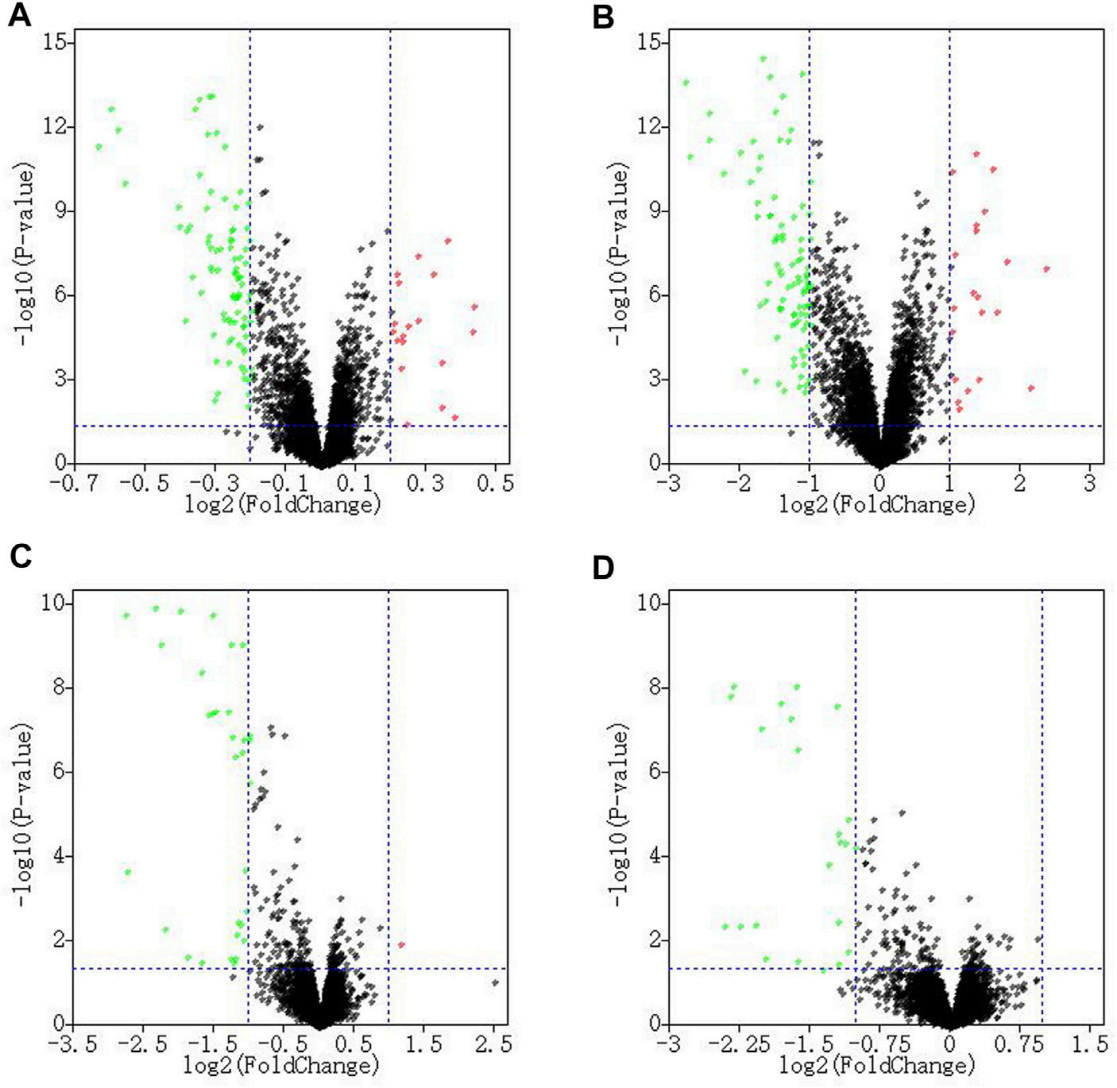
The volcano plot of DEGs. The volcano plot shows the DEGs of the glomerulus GSE99339 **(A)** and GSE104948 **(B)**, and the renal tubule GSE99325 **(C)** and GSE104954 **(D)**.

Our research methodology roadmap is shown in Figure 1. In summary, gene expression profiles of GSE99325, GSE99339, GSE104948 and GSE104954 were obtained from GEO database. The information of those series is given in Supplementary Material 1. In glomerular samples compared with LN, the GSE99339 identified 19 up-regulated and 89 down-regulated DEGs. The GSE104948 series identified 19 up-regulated DEGs and 92 down-regulated DEGs. The two series shared 14 up-regulated DEGs and 77 down-regulated DEGs. In the renal tubule samples compared with LN, the GSE99325 series identified 1 up-regulated DEGs and 34 down-regulated DEGs. The GSE104954 series identified 0 up-regulated DEGs and 23 down-regulated DEGs. Two series shared and 21 down-regulated DEGs, and no shared up-regulated DEGs. The results are shown in Figure 1 and Supplementary Material 2. To further demonstrate the difference in DEGs expression between MN and LN, we drew a volcano map to display it (Figure 2).

### Functional Enrichment Analyses

**FIGURE 3:**
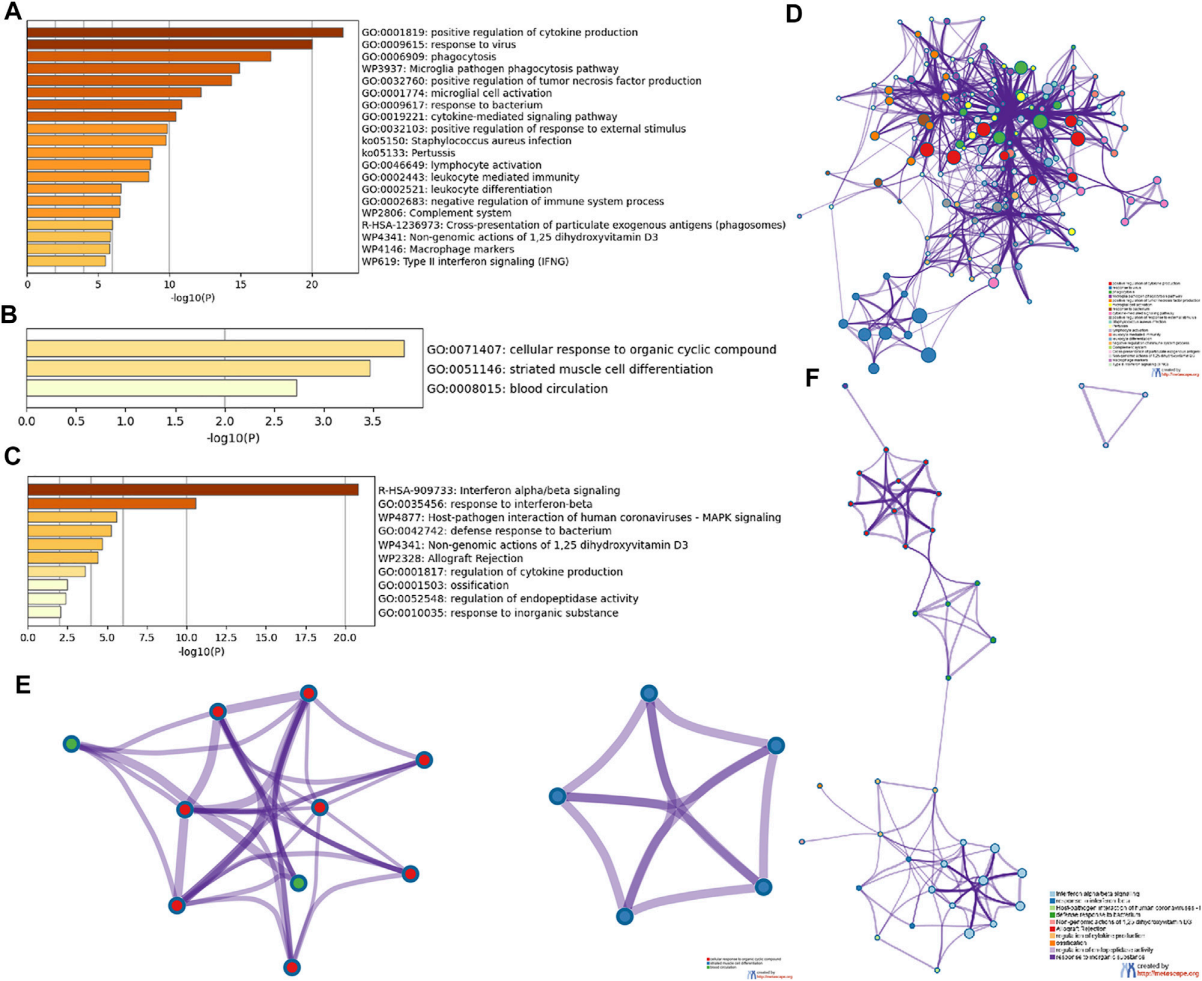
Detailed information related to changes in the biological function of DEGs was provided in the dataset by enrichment analysis. Using Metascape, we performed functional enrichment analysis of genes down-regulated **(A)** and up-regulated **(B)** in glomerulus, and down-regulated genes **(C)** in renal tubules. We then selected a representative subset of terms from the glomerular down-regulated gene cluster **(D)**, up-regulated gene cluster **(E)**, and renal tubules down-regulated gene cluster **(F)** and converted them into a network layout. Each term is represented by a circular node whose size is proportional to the number of inputs in the term, and whose color indicates its cluster identity.

Metascape enrichment analysis shows that glomerular sample from MN patients were down-regulated for positive regulation of cytokine production, response to virus, phagocytosis, microglia pathogen phagocytosis pathway, positive regulation of tumor necrosis factor production, microglial cell activation, response to bacterium, cytokine-mediated signaling pathway, positive regulation of response to external stimulus, lymphocyte activation, etc. Cellular response to organic cyclic compound, striated muscle cell differentiation, blood circulation were up-regulated in glomerular sample from MN patients. Interferon alpha/beta signaling, response to interferon-beta, host-pathogen interaction of human coronaviruses-MAPK signaling pathway, defense response to bacterium, non-genomic action of 1,25 dihydroxyvitamin D3, allograft rejection, regulation of cytokine production, ossification, regulation of endopeptidase activity, response to inorganic substance were down-regulated in renal tubule sample from MN patients (Figure 3). In terms of pathways, there are significantly enriched pathways in down-regulated DEGs in glomerular samples only. These pathways were enriched in *Staphylococcus aureus* infection, pertussis, complement and coagulation cascades, legionellosis, leishmaniasis, phagosome, toll-like receptor signaling pathway, tuberculosis, leukocyte transendothelial migration, measles. Although other DEGs can also obtain enrichment pathways, for example, the up-regulated DEGs in glomerular samples are enriched in nicotinate and nicotinamide metabolism, basal cell carcinoma, renin secretion, etc. Down-regulated DEGs in renal tubule samples are enriched in prion diseases, *S. aureus* infection, pertussi, etc. Those enrichment analysis results were not statistically significant (Supplementary Material 3). Enrichment results of biological process, Cellular Component and molecular function in GO were also shown in Supplementary Material 3. Compared to healthy controls, LN patients were also upregulated for interferon-beta production, regulation of response to biotic stimulus, immune effector process, negative regulation of endopeptidase activity, adaptive immune system. MN patients were also down-regulated for nuclear receptors meta-pathway, regulation of epithelial cell differentiation, multi-multicellular organism process, response to calcium ion, transcriptional misregulation in cancer, etc. The enrichment analysis results of DEGs obtained by comparing LN and MN with healthy controls were shown in Supplementary Material 4.

### PPI Network Construction and Identification of Hub Genes

**FIGURE 4:**
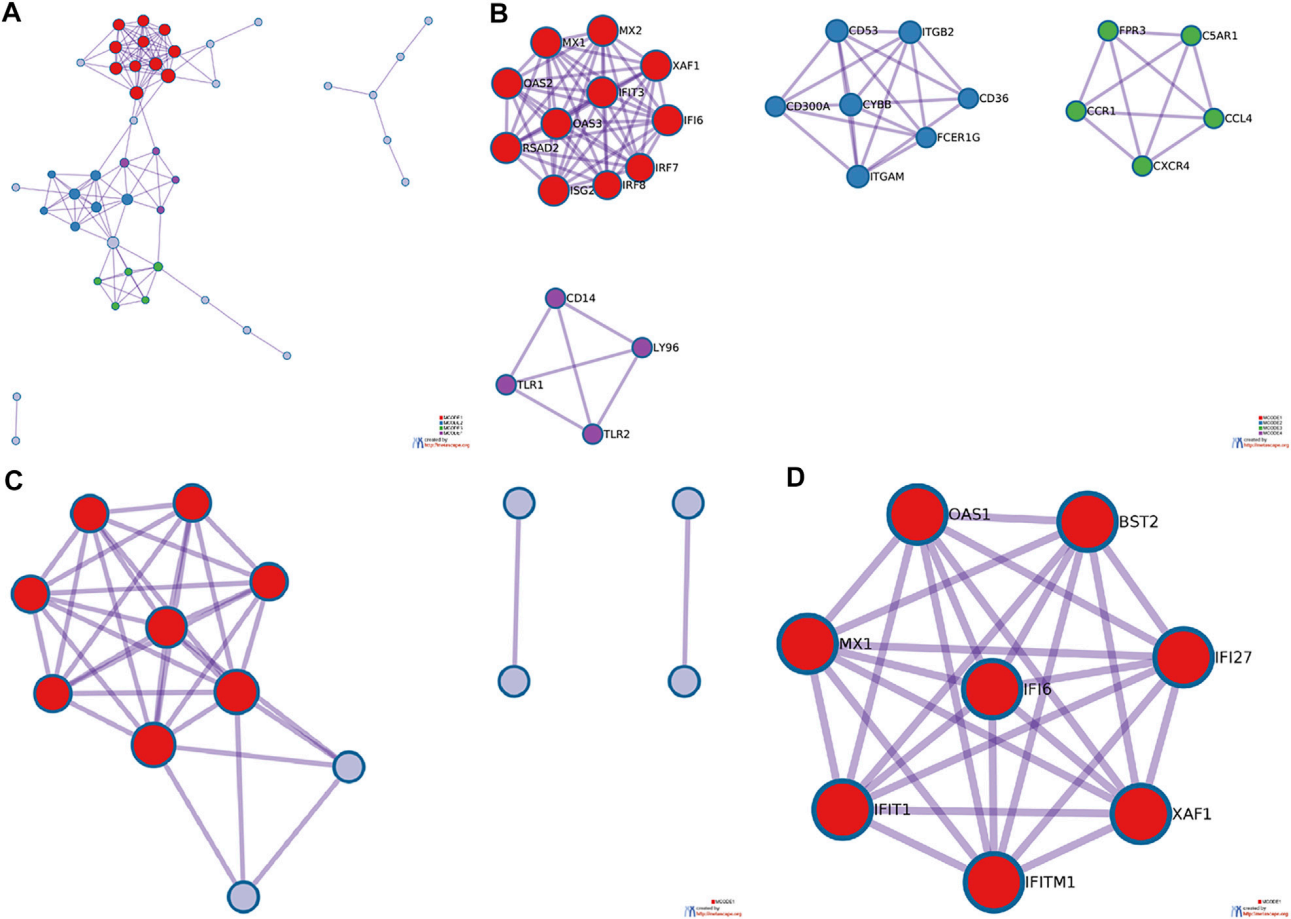
PPI network diagram of DEGs in glomerular and tubular. Metascape was used to construct the spatial distribution characteristics of the macroscopic PPI network model of glomerular DEGs **(A)** and renal tubule DEGs **(C)**, and the clusters of glomerular DEGs **(B)** and renal tubule DEGs **(D)** were selected.

Metascape was used to map the PPI network of glomerular and tubule DEGs. The four clusters in the glomerular PPI network map contain 27 genes Figures 4A,B. The meaning of MX dynamin like GTPase 1 (MX1), MX2, 2′-5′-oligoadenylate synthetase 2 (OAS2), interferon induced protein with tetratricopeptide repeats 3 (IFIT3), XIAP associated factor 1 (XAF1), radical S-adenosyl methionine domain containing 2 (RSAD2), OAS3, interferon alpha inducible protein 6 (IFI6), interferon stimulated exonuclease gene 20 (ISG20), interferon regulatory factor 8 (IRF8), IRF7 were interferon alpha/beta signaling, cytokine signaling in immune system and interferon signaling. The meaning of Cluster of differentiation 53 (CD53), integrin subunit beta 2 (ITGB2), CD300A, cytochrome b-245 beta chain (CYBB), CD36, integrin subunit alpha M (ITGAM), Fc fragment of IgE receptor Ig (FCER1G) were neutrophil degranulation, neutrophil activation and microglia pathogen phagocytosis pathway. The meaning of formyl peptide receptor 3 (FPR3), complement C5a receptor 1 (C5AR1), C-C motif chemokine receptor 1 (CCR1), C-C motif chemokine ligand 4 (CCL4), C-X-C motif chemokine receptor 4 (CXCR4) were peptide ligand-binding receptors, G alpha (i) signalling events and Class A/1 (Rhodopsin-like receptors). The meaning of CD14, lymphocyte antigen 96 (LY96), toll like receptor 1 (TLR1), TLR2 were MyD88 deficiency (TLR2/4), IRAK4 deficiency (TLR2/4) and regulation of TLR by endogenous ligand. Compared with MN, the above genes are highly expressed in LN. In other words, the above functions are stronger in the glomerulus of LN than in MN. The cluster in the renal tubule PPI network contains eight genes Figures 4C,D. The meaning of OAS1, bone marrow stromal cell antigen 2 (BST2), MX1, IFI6, IFI27, IFIT1, interferon induced transmembrane protein 1 (IFITM1), XAF1 were interferon alpha/beta signaling, interferon signaling and defense response to virus. Compared with MN, the above genes are highly expressed in LN. In other words, the above functions in the renal tubules of LN are stronger than those of MN. The three hub genes included in the two groups of data were MX1, IFI6 and XAF1. In addition to interferon alpha/beta signaling, the meaning of hub genes in LN also include ISG15 antiviral mechanism compared with healthy controls. The hub genes of LN and healthy controls are shown in Supplementary Material 5. Interestingly, interferon alpha/beta signaling was abnormal in both glomerular and tubule samples. That’s probably the key to the difference between MN and LN.

### Selection of Significantly Different Genes From the DEGs

**FIGURE 5:**
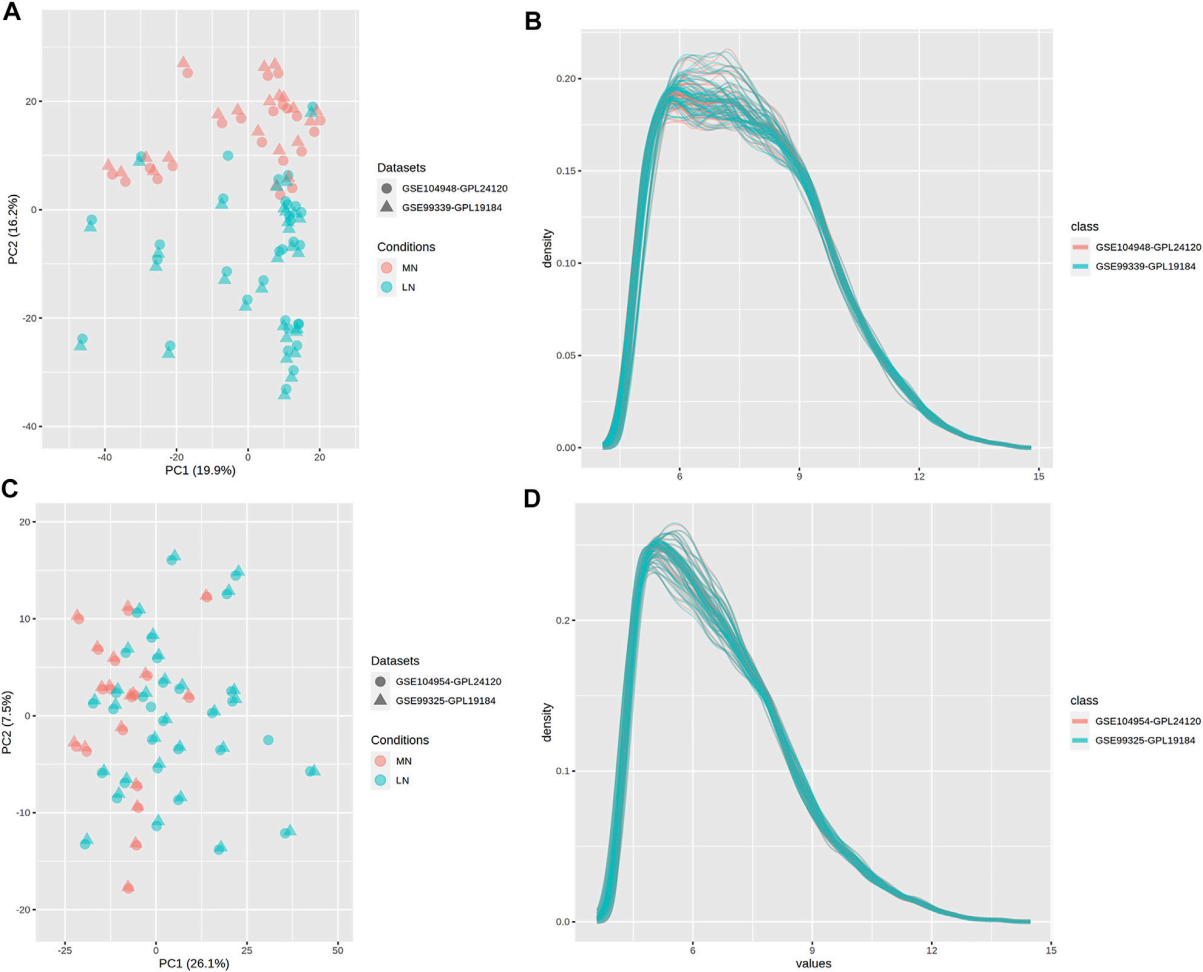
PCA plots and density plots. NetworkAnalyst was used to integrate gene expression profiles, and PCA plot **(A)** and density plot **(B)** of glomerulus, and PCA plot **(C)** and density plot **(D)** of renal tubules were drawn. The farther the distance between points or lines in the graph, the greater the difference between the suggested data.

**FIGURE 6:**
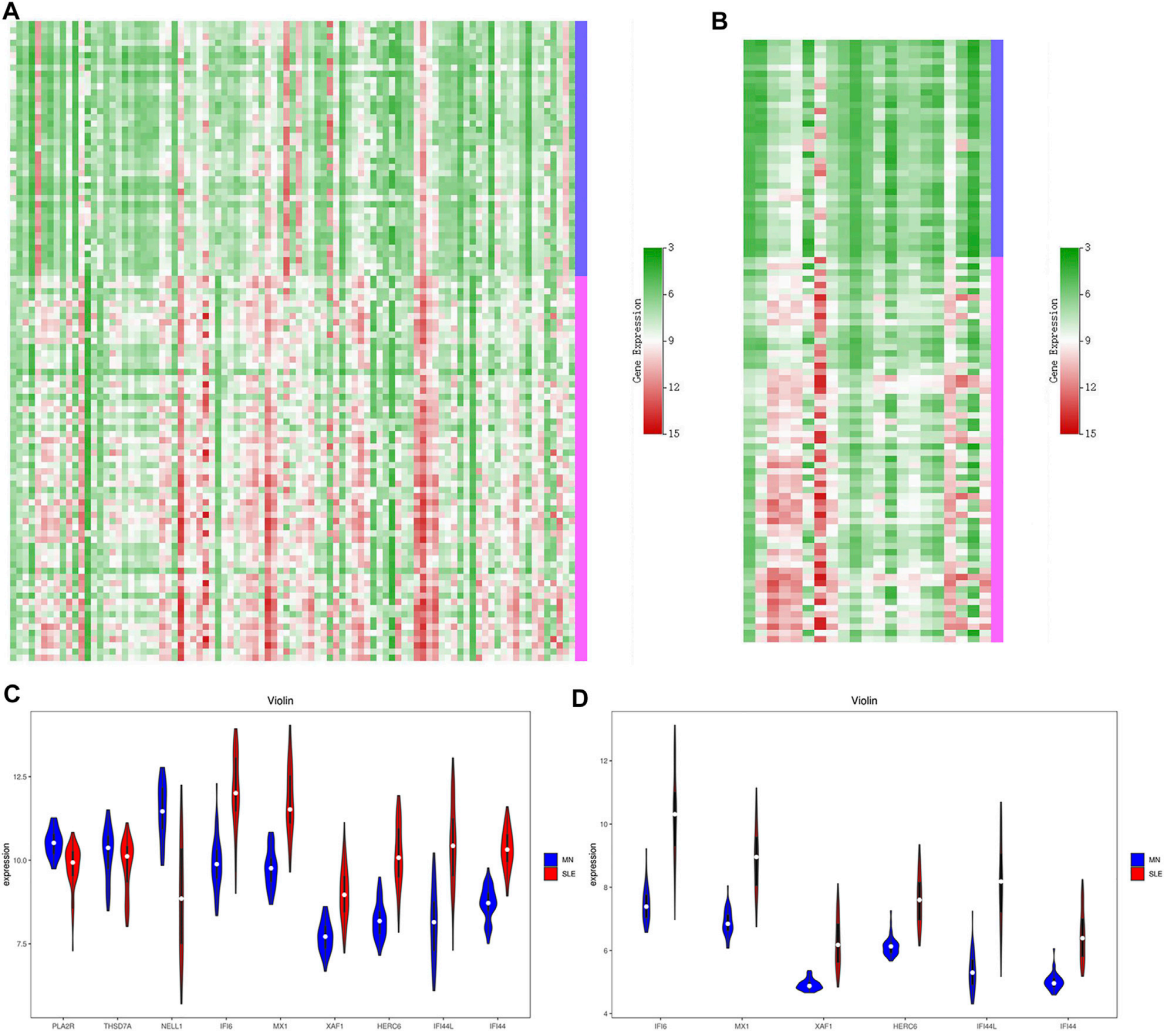
Heat maps and violin plots of DEGs. According to the above data integration results, the heat maps of glomerular DEGs **(A)** and renal tubule DEGs **(B)** were drawn. Green represents low expression and red represents high expression. We then screened out the most representative genes and mapped the violin plots of these genes in the glomerular sample **(C)** and these genes in the renal tubules **(D)**. Blue is MN and red is LN.

In addition, we searched for genes with the most significant expression differences in the patient’s kidney samples to search for the key biomarkers through enrichment analysis and PPI network mapping. We used the “Multiple Gene Expression Table” in Networkanalyst to integrate the gene expression profiles. We plotted PCA plots and density plots to show that the batch effect between data sets had been reduced (Figure 5). Based on the new gene expression profile, we selected three significantly different genes from the DEGs shared by glomeruli and renal tubules. Based on the new gene expression profile, we selected three significantly different genes, HERC6, IFI44 and IFI44L, from the DEGs shared by glomeruli and renal tubules. Based on the above data, we made heat maps of DEGs of glomerulus and renal tubules respectively to visually show the differences in gene expression levels (Figures 6A,B).

Based on the above results, six key biomarkers with potential diagnostic significance were summarized, namely IFI6, MX1, XAF1, HERC6, IFI44, and IFI44L. We used previously integrated data to draw the violin plots to show the differences between these genes (Figures 6C,D). In addition, We also selected MN specific pathogenic antigen phosphoesterase A2 receptor (PLA2R) from glomerular data. Serum spondin Domain Containing 7A (THSD7A) and Neural Epidermal Growth factor-like 1 (NELL1) were compared. However, of the three genes, only NELL1 was differentially expressed in the glomerulus, while PLA2R and THSD7A were not.

### Diagnose Signifcance of DEGs

**FIGURE 7:**
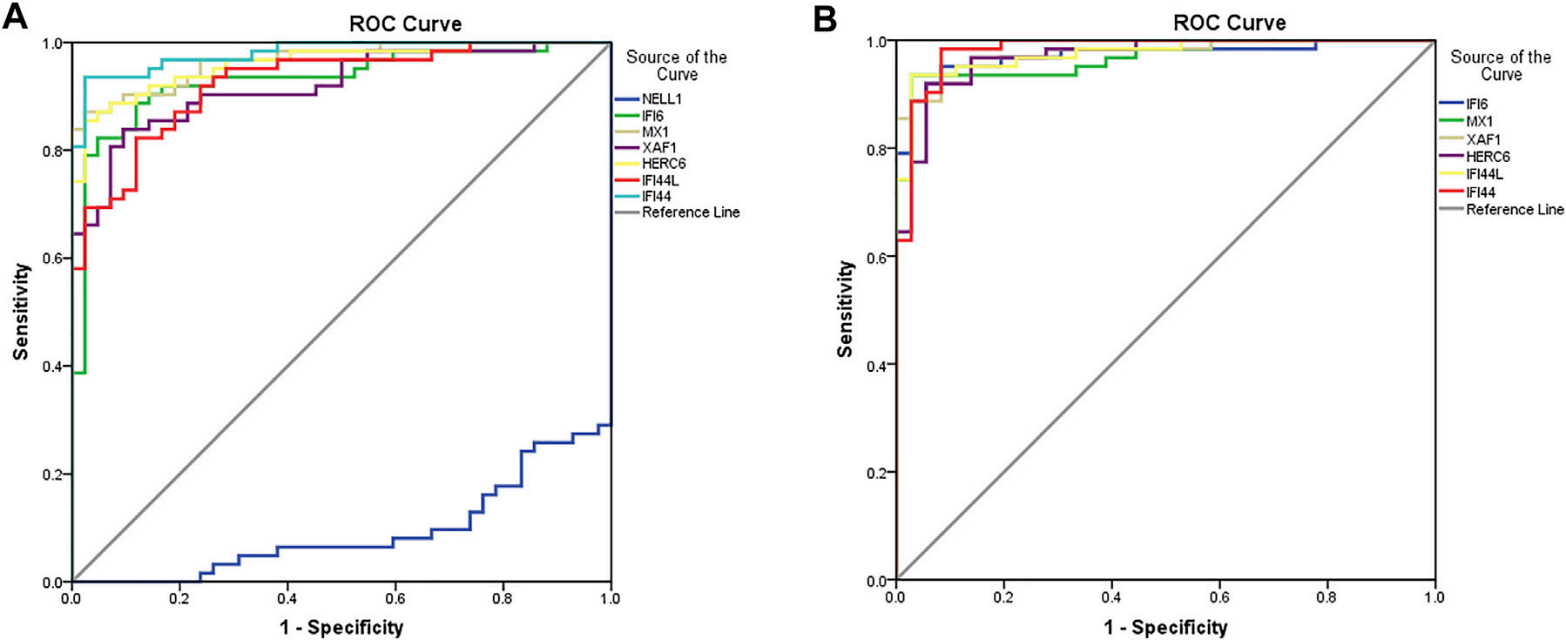
Diagnostic properties of genes. The diagnostic performance of these genes in glomerulus **(A)** and renal tubules **(B)** was calculated according to the gene expression levels in MN and SLE. AUC>0.95 indicates that the model has a good fitting effect.

Finally, in order to determine which DEGs have the most discriminative significance for MN and LN, ROC analysis was used to explore the sensitivity and specificity of DEGs for MN and LN (Figure 7). The results showed that IFI44 and MX1 had the best diagnostic value in differentiating MN patients from LN patients in glomerular group, with AUC of 0.980 and 0.967, respectively. NELL1 has no diagnostic value for distinguishing MN from LN. In the data of renal tubules, IFI44 and XAF1 have the best diagnostic value in distinguishing MN patients from LN patients, and their AUC is 0.982 and 0.976, respectively. The AUC of the remaining genes is shown in Supplementary Material 6.

## Discussion

We found three significant observations. First, the difference in Interferon alpha/beta signaling between MN and LN is the most significant. Second, MN specific pathogenic antigen expression in the glomerular was not significantly higher than LN. Third, interferon-induced proteins, as well as XAF1 and MX1, may be the key factors affecting patients’ response to air pollution.

Interferon (IFN) exerts antiviral effect after virus infection (Crow et al., 2019). It also affects cell differentiation, growth and immune regulation. It has three common types, IFN-α, IFN-β, and IFN-γ. Among them, IFN-α and IFN-β belong to type I IFN (Ng et al., 2016). Type I IFN is secreted by a variety type of cells, such as lymphocytes, macrophages, fibroblasts, endothelial cells, osteoblasts, and so on (Tailor et al., 2006; Crow et al., 2019; Kirchler et al., 2021). They play an antiviral role by stimulating macrophages and NK cells. They can activate JAK/STAT signaling pathway, and then regulate T cell proliferation and differentiation (Hosmillo et al., 2020). IFN-α is produced mainly by plasmacytoid dendritic cells (pDCs) and has therapeutic effect in a variety of human tumors and virus-induced diseases (Sisirak et al., 2013). IFN-α binds cell surface receptors and induces antiviral activity in a variety type of cells (Barchet et al., 2005; Agrawal and Gupta, 2011). IFN-α also activates regulatory B cells (Breg) (Menon et al., 2016; Dong et al., 2020). IFN-β is produced in large quantities by fibroblasts (Gao et al., 2021). They also have antiviral activity and are mainly involved in innate immune responses. IFN-β can inhibit the production of vascular endothelial growth factor (VEGF) to block tumor angiogenesis, and play an anti-tumor effect (Jablonska et al., 2010). IFN-β plays an important role in the survival and development of transitional B cells (de Laurentiis et al., 2015). IFN-β can reduce the recurrence rate of multiple sclerosis. (Petersen et al., 2018). Thus, IFN-α and IFN-β are closely related to autoimmunity. However, IFN-α and IFN-β can also play roles in the pathogenesis of LN by activating the autoimmune response and inflammatory response to aggravate the disease. IFN-α increased significantly in peripheral blood of patients with LN (Adel and Sadeq, 2020). IFN-α has been reported to accelerate kidney injury in LN (Wolf et al., 2018). IFN-α is also a common intervention for simulating podocyte injury *in vitro* (Qi et al., 2018; Zhou X et al., 2019). However, it has been reported that the decrease of IFN-β expression can indirectly alleviate the damage of TLR3 activator to glomerular endothelial cells (Hirono et al., 2020). In some cases, IFN-β therapy for relapsing-remitting multiple sclerosis (RRMS) has resulted in LN or thrombotic microangiopathy (Allinovi et al., 2017; Gianassi et al., 2019). Pegylated -IFN-α 2B treatment of chronic hepatitis C can lead to MN, while no recurrence of kidney disease was observed after stopping the treatment of IFN (Gianassi et al., 2019). However, this MN is not idiopathic, and recombinant IFN-α has been reported to have a therapeutic effect on Hepatitis B associated MN (Lin, 1995; Lopes Neto et al., 1998). These evidences are sufficient to suggest that IFN-α and IFN-β play important roles in the pathogenesis of LN, and this signaling is down-regulated in MN patients. This may indicate that IFN-α and IFN-β might be the key to affect the occurrence of LN or MN. In addition, there is extensive bioassay literature showing significant differences in LN patients’ own interferon alpha/beta signals from healthy controls (Der et al., 2017; Arazi et al., 2019; Der et al., 2019). However, it is clear from our results that LN differs from healthy controls in more than just interferon alpha/beta signaling. This is enough to prove that our conclusion is a certain specificity.

MN is thought to be caused by IgG that targets podocytes (Couser, 2017). In adults, the autoantigen of MN include PLA2R, THSD7A and NELL1, with detection rates of 70%–80%,3%–5% and 5–10% in MN patients, respectively (Beck et al., 2009; Tomas et al., 2014; Sethi et al., 2020). Among them, PLA2R is a member of the mannose receptor family of type C exogenous lectin superfamily, and it is a type I transmembrane protein, including long extracellular segment, transmembrane segment and short intracellular segment (Dong et al., 2017). It has shown that PLA2R inhibits inflammatory response by binding PLA2 protein, and the increase of its expression is related to inflammatory stimulation and aging (Augert et al., 2009; Griveau et al., 2018; Liu et al., 2019). This is also evidence that IMN is more common in the elderly. The incidence of the disease is associated with environmental pollution, or the patient’s history of pneumonia in adolescence (Bally et al., 2016). Anti-PLA2R antibody in peripheral blood is considered to be of diagnostic value for idiopathic MN, and its detection has been widely used in clinical diagnosis, prediction and guidance of treatment (de Vriese et al., 2017; Bobart et al., 2019). However, not all patients with positive serum anti-PLA2R antibodies are idiopathic MN. It has been reported that among 32 patients with secondary MN, 9 cases were positive for anti-PLA2R antibody, including 7 cases of cancer, 1 case of Crohn’s disease and 1 case of scleroderma (Radice et al., 2018). It has been reported that about 7% of patients are positive for anti-PLA2R antibody in serum but negative for PLA2R in renal tissue (Debiec and Ronco, 2011). There are also many cases of IMN recurrence after transplantation. Cuarental et al. increased PLA2R expression in renal podocytes by injecting TWEAK to 14-week-old C57/BL mice, but proteinuria was not reported in this study (Cuarental et al., 2020). Moreover, PLA2R is not a protein specifically expressed in the kidney. In addition to glomerular epithelial cells, PLA2R is also expressed in alveolar macrophages, neutrophils, placenta, liver and skeletal muscle (Ancian et al., 1995; Liu et al., 2020). Therefore, it is still doubtful whether the abnormal protein structure of kidney PLA2R or increased expression is the first factor leading to the MN. Our results can also support our view, because there is no statistically significant difference in the expression of PLA2R in the glomerular samples of MN and LN patients. Of the other two pathogenic autoantigens believed to be MN, THSD7A was initially described as an endothelial protein expressed in the placental vascular system that promotes endothelial cell migration during angiogenesis (Wang et al., 2010). NELL1 is a recently discovered autoantigen of IMN. It is highly expressed in osteoblasts and promotes bone regeneration. The C-terminal of NELL1 mediates osteoblast adhesion through integrin α3β2 (Hasebe et al., 2012). NELL1 also promotes bone formation through stem cell regeneration (Lee et al., 2021). Thus, these three autoantigens are functionally different. Many autoantigens with different functions would be discovered in the future. However, the more different their functions are, the more it indicates that the root cause of MN is not these autoantigens. It may be that the expression or function of some genes behind these antigens is abnormal. And in some special cases, these proteins become pathogenic autoantigens.

Six key biomarkers with potential identification value were IFI6, MX1, XAF1, HERC6, IFI44L, and IFI44, among which IFI6, MX1, and XAF1 were the hub genes that differentiated MN from LN. IFI6, or interferon alpha inducible protein 6, was originally identified as one of the interferon-induced genes. The interferon-stimulated genes can be upregulated by type I IFN, and has anti-apoptotic and antiviral effects (Park et al., 2013; Qi et al., 2015). It has been shown that IFI6 is upregulated in LN (Bing et al., 2016; Zhao et al., 2021). MX1, MX Dynamin like GTPase 1, is an important antiviral gene in human body (Spitaels et al., 2019). IFN-β produced by TLR3 or TLR4 stimulation can promote MX1 transcription (Nistler et al., 2020). Expect for LN, MX1 up-regulation also exists in rheumatoid arthritis, idiopathic interstitial pneumonia, alopecia areata and other autoimmune diseases (McDonagh and Tazi-Ahnini, 2002; Hamano et al., 2017; Yokoyama-Kokuryo et al., 2020). XAF1, also known as XIAP associated factor 1, plays an important role in cell apoptosis (Jiang et al., 2018). Besides LN, sjogren’s syndrome and multiple sclerosis have also seen abnormal expression of this gene (Serrano-Fernández et al., 2010; Yao et al., 2019). In addition to these hub genes, the most specific and sensitive is IFI44, or interferon inducible protein 44. It is upregulated in dermatomyositis, rheumatoid arthritis, sjogren’s syndrome, LN and other autoimmune diseases (Yao et al., 2019; Gaurav et al., 2021; Lerkvaleekul et al., 2021). Because IFI44 is overexpressed in head and neck squamous cell carcinoma cells and is significantly associated with clinical outcome, it is considered a potential prognostic indicator of this disease (Pan et al., 2020). However, the expression of these genes is down-regulated in MN. This may indicate that the pathogenesis of MN may be different from common autoimmune diseases. Exploring its mechanism requires some new ideas.

In addition, there are many limitations in our study. First of all, this study was analyzed based on GEO database. We can only observe differences in gene expression, not accurately reflect changes at the protein level. This makes our conclusions not entirely accurate. Therefore, further basic experiments are needed to confirm our results. Second, we could not categorize those data. The severity of the disease, the pathological type of LN, and the autoantigen of MN would affect the gene expression in glomerulus and renal tubules of patients. And that is what online database research could not figure out. Third, MN and LN are autoimmune diseases. It is not enough to analyze local changes in the kidney. We also need to detect gene expression in peripheral blood mononuclear cells (PBMCs) to fully explore the differences in molecular mechanisms and key biomarkers between the two diseases.

According to the MN pathogenesis hypothesis that extracellular traps lead to autoantigen exposure, patients are more likely to get LN or antineutrophil cytoplasmic antibody (ANCA) associated vasculitis in this situation. However, air pollution has also been linked to autoimmune diseases such as rheumatoid arthritis, multiple sclerosis, type 1 diabetes, and even autism (Lanzinger et al., 2018; Yousefian et al., 2018; Zhao et al., 2019; Di et al., 2020; Noorimotlagh et al., 2021). Therefore, although this hypothesis has pioneering significance for the research direction of MN, it is relatively lack of specificity. In addition, animal models of LN by stimulating the respiratory system are relatively well established (Pfau et al., 2008; Guo et al., 2016). This will make preliminary preparation for the preparation of gene knockout MN animal model in the next stage. However, the AUC of IFI6, MX1 and XAF1 as hub genes are all >0.95. Therefore, combined knockout can be attempted when necessary to observe the effect on animal models. In order to develop a MN animal model that is closer to human pathogenesis.

## Data Availability

The original contributions presented in the study are included in the article/Supplementary Material, further inquiries can be directed to the corresponding authors.

## Supplementary Material

Supplementary Material 1 Characteristics of the GSE99325, GSE99339, GSE104948 and GSE104954 datasets.

Supplementary Material 2 The shared DEGs in the GSE99325, GSE99339, GSE104948 and GSE104954.

Supplementary Material 3 DEGs enrichment results. GO enrichment in glomerular up-regulated (A), down-regulated (C), and tubule down-regulated (E) DEGs, as well as KEGG enrichment in glomerular up-regulated (B), down-regulated (D), and tubule down-regulated (F) DEGs are shown here.

Supplementary Material 4 DEGs enrichment results compared to healthy controls. Using Metascape, enrichment result in DEGs with LN patients (A) and in MN patients (B) are shown here.

Supplementary Material 5 PPI networks compared to healthy controls. Using Metascape, the hub genes in DEGs upregulated in LN patients compared with healthy controls are shown here.

Supplementary Material 6 Data of AUC in ROC.
