# Supplementary figures and images for: Exploring the Differences in Molecular Mechanisms and Key Biomarkers Between Membranous Nephropathy and Lupus Nephritis Using Integrated Bioinformatics Analysis
Source: Front Genet. 2022 Jan 3;12:770902. doi: 10.3389/fgene.2021.770902 (PMC8762271; doi:10.3389/fgene.2021.770902)

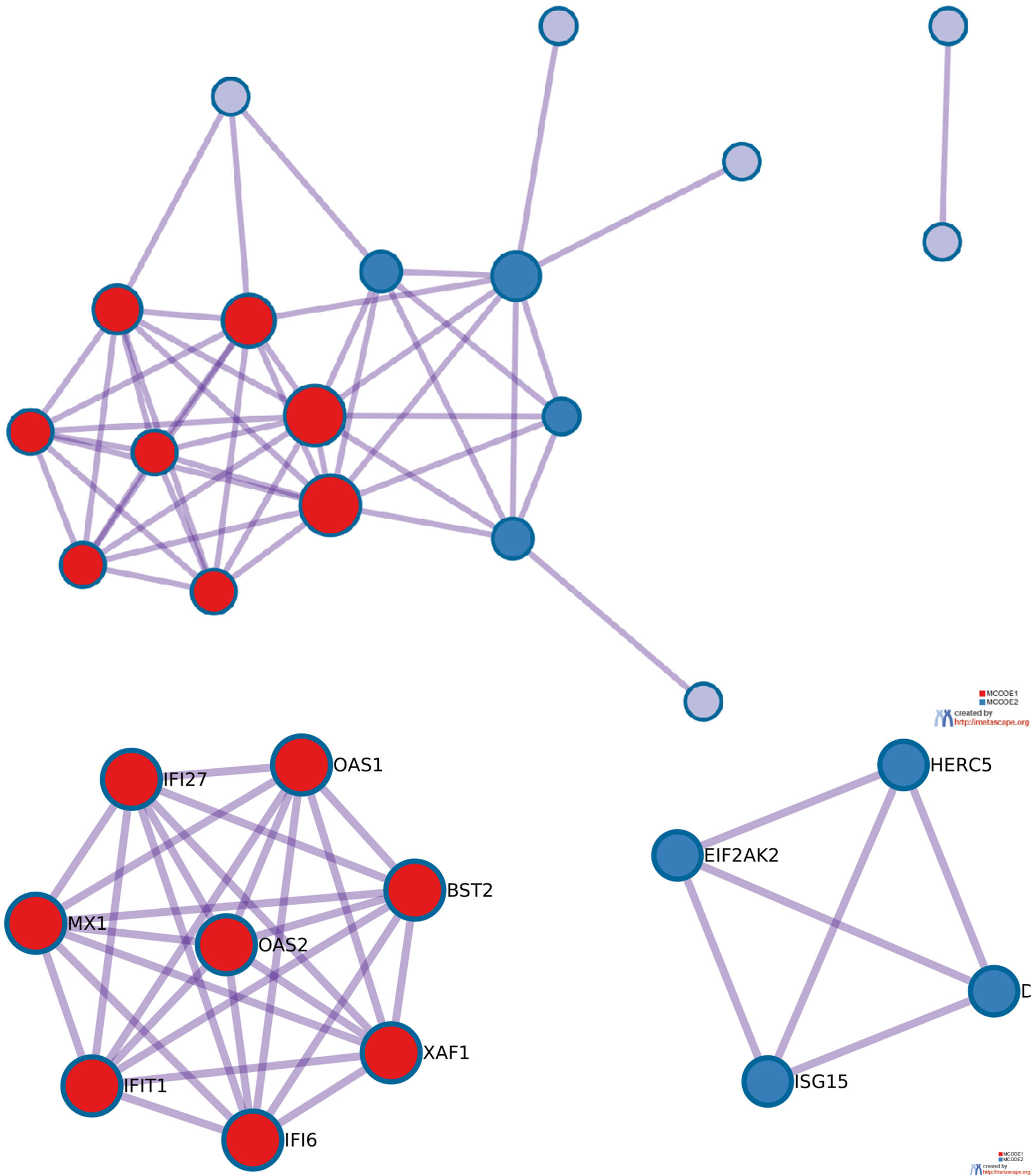

Supplement: Supplementary file 1 [file Image3.TIF]

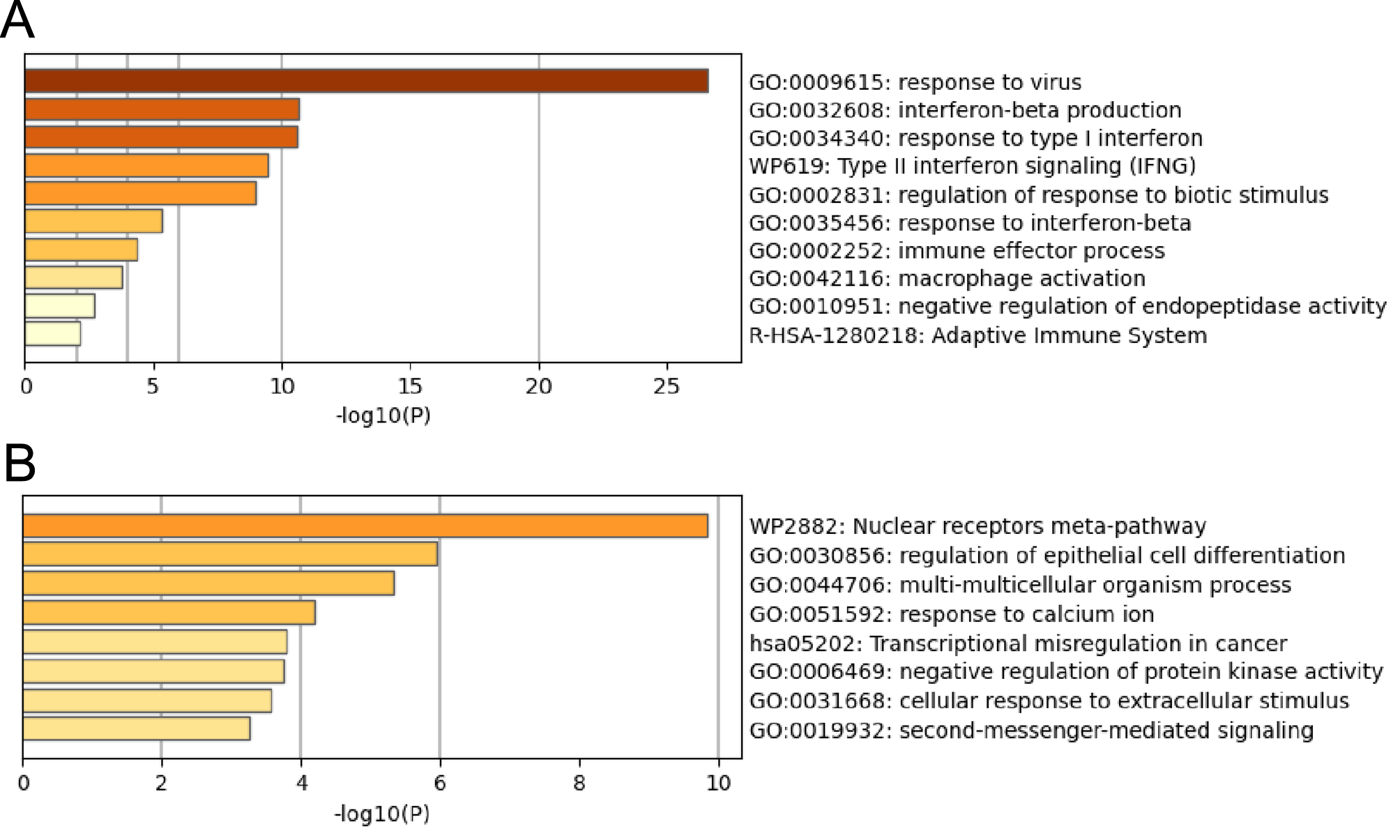

Supplement: Supplementary file 3 [file Image2.TIF]

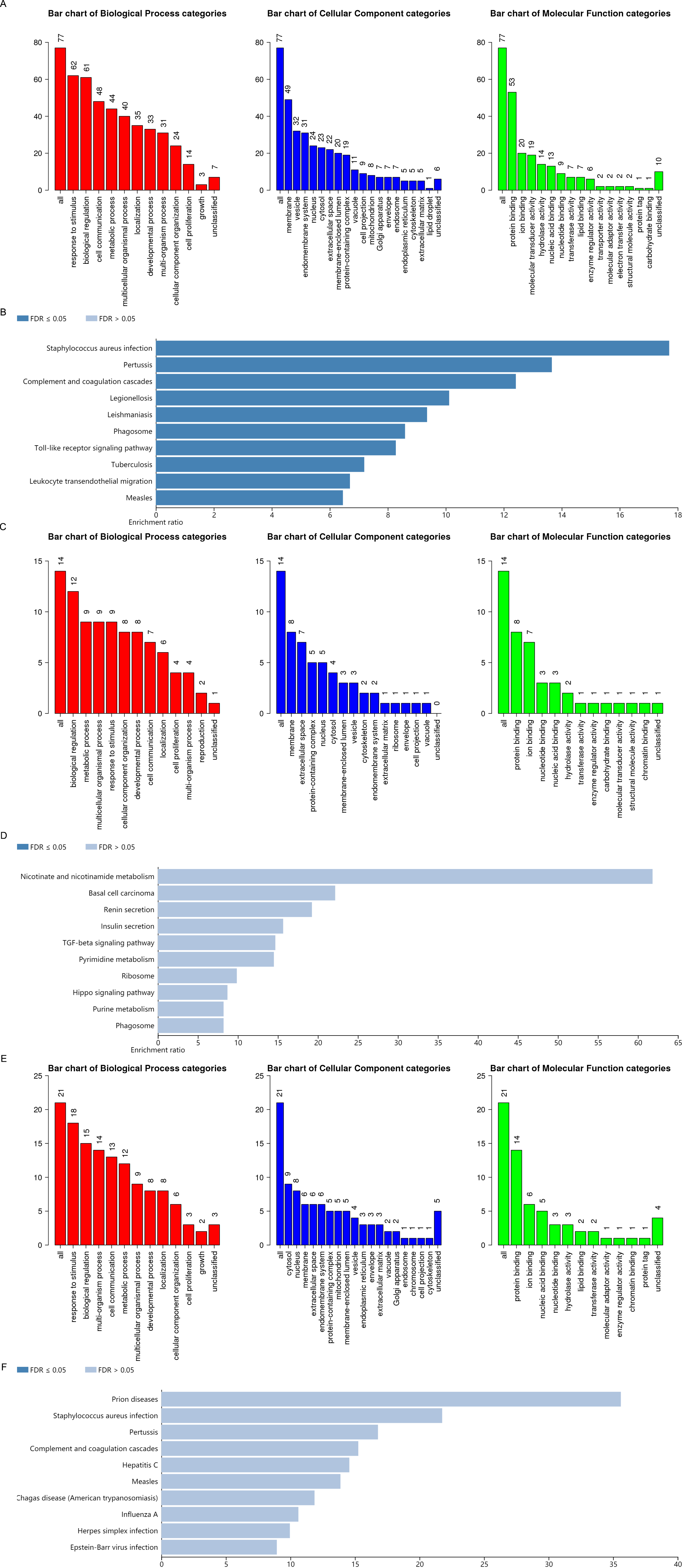

Supplement: Supplementary file 4 [file Image1.TIF]
