# Supplementary material for: Exploring the Differences in Molecular Mechanisms and Key Biomarkers Between Membranous Nephropathy and Lupus Nephritis Using Integrated Bioinformatics Analysis
Source: Front Genet. 2022 Jan 3;12:770902. doi: 10.3389/fgene.2021.770902 (PMC8762271; doi:10.3389/fgene.2021.770902)
Supplement: Supplementary file 2 [file DataSheet3.docx]

| Area Under the Curve (Glomerulus) | |
| --- | --- |
| Test Result Variable(s) | Area |
| NELL1 | 0.091 |
| IFI6 | 0.931 |
| MX1 | 0.967 |
| XAF1 | 0.920 |
| HERC6 | 0.961 |
| IFI44L | 0.925 |
| IFI44 | 0.980 |
|  |  |
| Area Under the Curve (Tubules) | |
| Test Result Variable(s) | Area |
| IFI6 | 0.972 |
| MX1 | 0.966 |
| XAF1 | 0.976 |
| HERC6 | 0.970 |
| IFI44L | 0.975 |
| IFI44 | 0.982 |
