# Supplementary material for: Exploring the Differences in Molecular Mechanisms and Key Biomarkers Between Membranous Nephropathy and Lupus Nephritis Using Integrated Bioinformatics Analysis
Source: Front Genet. 2022 Jan 3;12:770902. doi: 10.3389/fgene.2021.770902 (PMC8762271; doi:10.3389/fgene.2021.770902)
Supplement: Supplementary file 5 [file DataSheet2.docx]

| Position | Gene | Regulation | Gene title |
| --- | --- | --- | --- |
| Glomerulus | CDH12 | Up | cadherin 12 |
|  | MYLK3 | Up | myosin light chain kinase 3 |
|  | INSL4 | Up | insulin like 4 |
|  | NT5E | Up | 5'-nucleotidase ecto |
|  | BMP2 | Up | bone morphogenetic protein 2 |
|  | DDX3Y | Up | DEAD-box helicase 3 Y-linked |
|  | COLEC12 | Up | collectin subfamily member 12 |
|  | RPS4Y1 | Up | ribosomal protein S4 Y-linked 1 |
|  | NELL1 | Up | neural EGFL like 1 |
|  | KDM5D | Up | lysine demethylase 5D |
|  | MYOZ2 | Up | myozenin 2 |
|  | SRPX2 | Up | sushi repeat containing protein X-linked 2 |
|  | ADCYAP1 | Up | adenylate cyclase activating polypeptide 1 |
|  | CHGA | Up | chromogranin A |
|  | TYROBP | Down | transmembrane immune signaling adaptor TYROBP |
|  | SAMSN1 | Down | SAM domain, SH3 domain and nuclear localization signals 1 |
|  | MS4A6A | Down | membrane spanning 4-domains A6A |
|  | CSF1R | Down | colony stimulating factor 1 receptor |
|  | IFIH1 | Down | interferon induced with helicase C domain 1 |
|  | MS4A4A | Down | membrane spanning 4-domains A4A |
|  | TLR1 | Down | toll like receptor 1 |
|  | PTPRC | Down | protein tyrosine phosphatase receptor type C |
|  | TLR2 | Down | toll like receptor 2 |
|  | AQP9 | Down | aquaporin 9 |
|  | BCL2A1 | Down | BCL2 related protein A1 |
|  | AOAH | Down | acyloxyacyl hydrolase |
|  | IFI44 | Down | interferon induced protein 44 |
|  | VSIG4 | Down | V-set and immunoglobulin domain containing 4 |
|  | MNDA | Down | myeloid cell nuclear differentiation antigen |
|  | CXCR4 | Down | C-X-C motif chemokine receptor 4 |
|  | OAS3 | Down | 2'-5'-oligoadenylate synthetase 3 |
|  | IGKC | Down | immunoglobulin kappa constant |
|  | IFIT3 | Down | interferon induced protein with tetratricopeptide repeats 3 |
|  | SRPX | Down | sushi repeat containing protein X-linked |
|  | GUSBP11 | Down | GUSB pseudogene 11 |
|  | CYTIP | Down | cytohesin 1 interacting protein |
|  | IL10RA | Down | interleukin 10 receptor subunit alpha |
|  | HCK | Down | HCK proto-oncogene, Src family tyrosine kinase |
|  | IFI44L | Down | interferon induced protein 44 like |
|  | ADAP2 | Down | ArfGAP with dual PH domains 2 |
|  | DOCK2 | Down | dedicator of cytokinesis 2 |
|  | IRF8 | Down | interferon regulatory factor 8 |
|  | CPVL | Down | carboxypeptidase vitellogenic like |
|  | IFI30 | Down | IFI30 lysosomal thiol reductase |
|  | CD14 | Down | CD14 molecule |
|  | FCER1G | Down | Fc fragment of IgE receptor Ig |
|  | NCF2 | Down | neutrophil cytosolic factor 2 |
|  | ISG15 | Down | ISG15 ubiquitin like modifier |
|  | HERC5 | Down | HECT and RLD domain containing E3 ubiquitin protein ligase 5 |
|  | THEMIS2 | Down | thymocyte selection associated family member 2 |
|  | FPR3 | Down | formyl peptide receptor 3 |
|  | LCP1 | Down | lymphocyte cytosolic protein 1 |
|  | CCR1 | Down | C-C motif chemokine receptor 1 |
|  | CYBB | Down | cytochrome b-245 beta chain |
|  | MX1 | Down | MX dynamin like GTPase 1 |
|  | IRF7 | Down | interferon regulatory factor 7 |
|  | OAS2 | Down | 2'-5'-oligoadenylate synthetase 2 |
|  | C5AR1 | Down | complement C5a receptor 1 |
|  | CD163 | Down | CD163 molecule |
|  | HERC6 | Down | HECT and RLD domain containing E3 ubiquitin protein ligase family member 6 |
|  | CD36 | Down | CD36 molecule |
|  | COL6A3 | Down | collagen type VI alpha 3 chain |
|  | CORO1A | Down | coronin 1A |
|  | LYZ | Down | lysozyme |
|  | C1QB | Down | complement C1q B chain |
|  | LAPTM5 | Down | lysosomal protein transmembrane 5 |
|  | CD300A | Down | CD300a molecule |
|  | C1QA | Down | complement C1q A chain |
|  | ITGB2 | Down | integrin subunit beta 2 |
|  | LY96 | Down | lymphocyte antigen 96 |
|  | ISG20 | Down | interferon stimulated exonuclease gene 20 |
|  | ITGAM | Down | integrin subunit alpha M |
|  | GPR65 | Down | G protein-coupled receptor 65 |
|  | XAF1 | Down | XIAP associated factor 1 |
|  | APOBEC3A | Down | apolipoprotein B mRNA editing enzyme catalytic subunit 3A |
|  | CSTA | Down | cystatin A |
|  | RNASE6 | Down | ribonuclease A family member k6 |
|  | FCN1 | Down | ficolin 1 |
|  | IFI6 | Down | interferon alpha inducible protein 6 |
|  | NNMT | Down | nicotinamide N-methyltransferase |
|  | RGCC | Down | regulator of cell cycle |
|  | CD53 | Down | CD53 molecule |
|  | C3AR1 | Down | complement C3a receptor 1 |
|  | CD52 | Down | CD52 molecule |
|  | MX2 | Down | MX dynamin like GTPase 2 |
|  | TFEC | Down | transcription factor EC |
|  | CCL4 | Down | C-C motif chemokine ligand 4 |
|  | RSAD2 | Down | radical S-adenosyl methionine domain containing 2 |
|  | CTSS | Down | cathepsin S |
|  | EVI2A | Down | ecotropic viral integration site 2A |
|  | EVI2B | Down | ecotropic viral integration site 2B |
| Tubules | IFITM1 | Down | interferon induced transmembrane protein 1 |
|  | JCHAIN | Down | joining chain of multimeric IgA and IgM |
|  | IFI27 | Down | interferon alpha inducible protein 27 |
|  | OAS1 | Down | 2'-5'-oligoadenylate synthetase 1 |
|  | MX1 | Down | MX dynamin like GTPase 1 |
|  | IGKC | Down | immunoglobulin kappa constant |
|  | GUSBP11 | Down | GUSB pseudogene 11 |
|  | SRGN | Down | serglycin |
|  | BST2 | Down | bone marrow stromal cell antigen 2 |
|  | XAF1 | Down | XIAP associated factor 1 |
|  | STAT1 | Down | signal transducer and activator of transcription 1 |
|  | IFI44L | Down | interferon induced protein 44 like |
|  | IFI6 | Down | interferon alpha inducible protein 6 |
|  | HERC6 | Down | HECT and RLD domain containing E3 ubiquitin protein ligase family member 6 |
|  | IFIT1 | Down | interferon induced protein with tetratricopeptide repeats 1 |
|  | S100A8 | Down | S100 calcium binding protein A8 |
|  | IFI44 | Down | interferon induced protein 44 |
|  | IGLL3P | Down | immunoglobulin lambda like polypeptide 3, pseudogene |
|  | C1QB | Down | complement C1q B chain |
|  | ISG15 | Down | ISG15 ubiquitin like modifier |
|  | C1QA | Down | complement C1q A chain |
