# Supplementary material for: Exploring the Differences in Molecular Mechanisms and Key Biomarkers Between Membranous Nephropathy and Lupus Nephritis Using Integrated Bioinformatics Analysis
Source: Front Genet. 2022 Jan 3;12:770902. doi: 10.3389/fgene.2021.770902 (PMC8762271; doi:10.3389/fgene.2021.770902)
Supplement: Supplementary file 6 [file DataSheet1.docx]

| Series code | Series geo accession | Series type | Number of samples | Group | Organism | Series platform id |
| --- | --- | --- | --- | --- | --- | --- |
| G1 | GSE99325 | Expression profiling by array | 48 | MN (n=18)  LN (n=30) | Homo sapiens | GPL19184 |
| G2 | GSE99339 | Expression profiling by array | 51 | MN (n=21)  LN (n=30) | Homo sapiens | GPL19184 |
| G3 | GSE104948 | Expression profiling by array | 53 | MN (n=21)  LN (n=32) | Homo sapiens | GPL24120 |
| G4 | GSE104954 | Expression profiling by array | 50 | MN (n=18)  LN (n=32) | Homo sapiens | GPL24120 |
